# Automated Detection and Characterization of Colon Cancer with Deep Convolutional Neural Networks

**DOI:** 10.1155/2022/5269913

**Published:** 2022-08-24

**Authors:** Md Imran Hasan, Md Shahin Ali, Md Habibur Rahman, Md Khairul Islam

**Affiliations:** ^1^Department of Computer Science and Engineering, Islamic University, Kushtia 7003, Bangladesh; ^2^Department of Biomedical Engineering, Islamic University, Kushtia 7003, Bangladesh

## Abstract

Colon cancer is a momentous reason for illness and death in people. The conclusive diagnosis of colon cancer is made through histological examination. Convolutional neural networks are being used to analyze colon cancer via digital image processing with the introduction of whole-slide imaging. Accurate categorization of colon cancers is necessary for capable analysis. Our objective is to promote a system for detecting and classifying colon adenocarcinomas by applying a deep convolutional neural network (DCNN) model with some preprocessing techniques on digital histopathology images. It is a leading cause of cancer-related death, despite the fact that both traditional and modern methods are capable of comparing images that may encompass cancer regions of various sorts after looking at a significant number of colon cancer images. The fundamental problem for colon histopathologists is differentiating benign from malignant illnesses to having some complicated factors. A cancer diagnosis can be automated through artificial intelligence (AI), enabling us to appraise more patients in less time and at a decreased cost. Modern deep learning (MDL) and digital image processing (DIP) approaches are used to accomplish this. The results indicate that the proposed structure can accurately analyze cancer tissues to a maximum of 99.80%. By implementing this approach, medical practitioners will establish an automated and reliable system for detecting various forms of colon cancer. Moreover, CAD systems will be built in the near future to extract numerous aspects from colonoscopic images for use as a preprocessing module for colon cancer diagnosis.

## 1. Introduction

According to the World Health Organization (WHO), cancer is the largest purpose of morality loss in the world[1]. Colon cancer develops in the large entrails (colon) or the rectum (end of the colon) [2]. Moreover, cancer is a broad term that surrounds various disorders in which peculiar cells originate inside the human body due to random mutations. These cells divide uncontrollably upon creation and disseminate throughout the organs. Most varieties of cancer, if left untreated, can eventually kill people. Cancer is the second prominent cause of mortality globally, behind cardiovascular illnesses, which is the biggest purpose of death worldwide, accounting for roughly 10 million deaths in 2020, as reported by the WHO [3]. Colon and rectal cancers are uncommon in underdeveloped countries but are the second-highest common type of cancer in rich societies. Each year, more than 940,000 instances appear worldwide, and approximately 500,000 people die due to colon cancer [4]. The incidence of malignant tumors has been increasing worldwide, attributable to population growth. It can affect any age group and is most frequently detected in the senior age group of 50–60 years [5]. By 2035, cancer mortality will be anticipated to reach 60% [6].

It typically begins forming small, benign collections of cells called polyps on the colon's inner wall. Some of these polyps may eventually grow into colon malignancies. A tumor originates in the majority of cases of colon cancer when healthy cells in the colon or rectum multiply uncontrollably. Adenocarcinoma of the colon or rectum begins in the epithelial cells of the large intestine and subsequently spreads to the other layers. Mucinous adenocarcinomas and signet ring cell adenocarcinomas are two distinctive yet aggressive forms of adenocarcinoma. Changes in one's physique over time are dependent on characteristics such as gender, ethnic origin, age, smoking habits, and socioeconomic status. However, alterations can occur within a few months if a person has a unique genetic syndrome. In rare instances, an individual gets the faulty gene responsible for cancer from sufferer parents. Individuals that are at action of acquiring hereditary malignancies should undergo routine screenings. These diagnostic procedures are expensive, and many people are unable to pay for them. Around 70% of cancer, fatalities occur in below and average-income nations [3]. According to 2016 data, just 26% of low-income nations have pathology aid essential to detect cancer on hand to the people; rich countries might provide diagnosis and analysis to more than 90% of their community [3]. Not just cancer, but a lack of appropriate medication leaves communities in developing and rising nations more vulnerable to a wide range of illnesses. To address this issue, these countries must invest extensively in public health, establish multiple laboratories and pathology centers equipped with the appropriate technology, and train additional personnel to perform diagnostic operations. Additionally, they must keep the costs of these tests within reach of persons living below the poverty line [3]. To be sure, none of these purposes are easy to accomplish for any country around the globe, and even if they are, they will not occur overnight. To maintain relevance in the treatment of cancers and to give these patients a realistic chance of survival, we must investigate alternative diagnostic approaches.

A potential clarification to this dilemma has come from a discipline entirely unrelated to medicine and healthcare [7–9]. In comparison with other fields of science and technology, computer science has arguably progressed the most in the previous 50 years. Machine learning (ML) offers a broad area of applications in pathology, from disease identification to intelligent systems that can recommend traditional medications based on a patient's symptoms [10].

The current way of detecting cancer is extremely time-consuming and labor-expensive. Pathologists must get extensive knowledge by studying labeled histopathological images to identify colon images. As a result, a significant amount of resources and manual work are squandered. As a result, increased diagnostic accuracy and diagnostic speed are necessary.

Computer technologies have garnered considerable attention due to their inherent advantages, including computational power, speed, and storage capacity. Researchers' focus has shifted to develop an automated method for cancer detection (prostate cancer [11], breast cancer [12], etc.) based on computer-aided diagnosis. ML [13] is one of the most exciting uses of computer-aided technology, owing to its capacity for human-like learning, which automatically improves the predictive performance of its models by learning from data. There have been numerous research studies [14, 15] conducted to date on colon cancer analysis helped by computer technologies. However, the particular systems are quite sophisticated in comparison.

In a large bound of applications, deep learning algorithms for image identification have proven to be incredibly effective, frequently outperforming human ability. The key concept is that an adaptable software network may be trained, parameters assigned values, to identify images through many tagged images. Once trained, the network can be used to classify the appropriate label for unlabeled images [16].

The current study's primary objective is to evaluate the use of deep learning for the histological investigation of colon cancer by analyzing digitized pathology images and resolving the effect of the suggested DCNN model [17–19]. With more minor preprocessing required than other classification algorithms, the algorithm's architecture is inspired by patterns of neurons and their connectivity within the human brain. The ability of the algorithm is to learn characteristics that outperform the rudimentary way of hand-engineering filters. The suggested model accepts input images with weights (learnable weights and biases) assigned to several features in the image and can discriminate between them. We use histopathology [20] slides as a dataset because the preparation method preserves the underlying tissue architecture and so provides an interdisciplinary image of disease and its influence on tissues. This study developed a finely tuned DL model capable of recognizing malignancies and organs in medical data. In addition, it can also revolutionize the entire healthcare landscape and has been utilized to identify diseases and successfully classify image samples.

The main contribution of our article is described as follows:

- We propose a fine-tuned DL model that yields promising results in the classification of colon cancer.
- Our proposed DL model achieves much better accuracy when compared to other existing related works within a short time.
- The proposed model can save both time and space by utilizing effective data processing techniques.

The following section organizes the paper: Section 2 discusses previously conducted research in the current domain.  Section 3 represents an overview of the LC25000 dataset and the methodology.  Section 4 provides an overview of our proposed DCNN model with its architecture and training parameters.  Section 5 summarizes all experimental findings and results. Finally, Section 6 summarizes our experiment and offers some recommendations for further research.

## 2. Related Work

Lee Lusted saw the potential of computers in medical diagnosis for the first time in 1955 [21]. In 1963, a research study established the first practical application of computers in medical picture diagnosis [22]. Histopathology is a rapidly growing field, and histopathological images are becoming more prevalent. With the introduction and affordability of whole-slide digital scanners, tissue histology slides can now be digitized and preserved as digital images [20]. Eesa and Arabo [23] reported an early investigation in which data for the exploration were gathered via micrography and analyzed using a multivariate analytic approach. As indicated in Esgiar's samples' low resolution, the drawback is that the information content is restricted to a low grade. Throughout the 1970s and 1980s, one of the most explored CAD applications was lung cancer detection utilizing chest radiographs. However, the apparatus of the deep learning (DL) approaches fundamentally altered the field. Analysts have applied DL and non-deep learning-based learning algorithms to practically every sort of cancer detection. Due to the fact that our work relates to lung and colon cancer diagnosis, we will address the described approaches in these two areas in detail. The images and the techniques used to process those images differ between these approaches, the types of characteristics collected, and the architecture of the ML model used to identify cancer [24, 25]. Shi et al. [26] recommended a method for lung cancer diagnosis using multimodal sparse representation-based classification (mSRC) in 2013. Jin [27] suggested a computer-aided diagnosis (CAD) method for lung cancer classification in 2014, based on the inquiry of CT scan data. Their study analyzed needle biopsy specimens and automatically classified lung cancer by automatically segmenting 4372 cell nuclei regions. On average, their system achieved a classification accuracy of 88.10%. Xu et al. extracted four sorts of features from a sample of histology colon images and categorized them using three distinct types of support vector machines (SVMs) [28]. The authors [29] developed a deep CNN model to differentiate the cancer tissue component of colon cancer using whole-slide pathological images from The Cancer Genome Atlas (TCGA) of 164 patients. The predictive value of the peri-tumoral stroma (PTS) score for LNM was determined to be 0.038. Three CNN architecture variants (ResNet-18, ResNet-30, and ResNet-50) were used in this study to classify digitized images of colonic tissue. ResNet-50 had the best accuracy (93.91%), followed by ResNet-30 and ResNet-18, both of which had a 93.04% accuracy [30]. As a solution to the sparse labeling of histopathology images, CNN models [31] are used to evaluate images of colon cells by implementing a multistep training technique [32]. In practice, gradient vanishing issues limit traditional CNN's capacity to offer generic, transferrable pathological data representations. Toraman et al. presented research in that used Fourier transform infrared (FTIR) spectroscopic signals to classify the likelihood of colon cancer [33]. GradCAM and SmoothGrad are also utilized to visualize the attention images of pretrained CNN models distinguishing cancerous and benign images, according to Nasser Esgiar [34].

Classification of cells and nuclei has been utilized for various histopathology-linked applications. Dalle et al. [35] graded nuclear pleomorphisms in breast cancer pictures using the shape, texture, and size of nuclei. Malon and Cosatto [36] used color, texture, and shape information to train a CNN classifier to distinguish mitotic and nonmitotic cells. Nguyen et al. [37] divided nuclei into cancer and normal nuclei based on their appearance and used the area of identified nuclei to identify cancer glands in prostate cancer. Shape features have also been employed to identify between normal and cancerous nuclei in prostate histology pictures using an unsupervised manifold learning framework [38]. Sharma et al. [39] proposed segmenting and classifying nuclei using the AdaBoost classifier and parameters such as intensity, morphology, and texture. The effort concentrated on nuclei segmentation, with little emphasis on classification performance. Abbas et al. [40] conducted a comparative investigation using CNNs to diagnose squamous cell carcinomas. It evaluates different CNN architectures, including AlexNet, VGG-16, and ResNet, and achieves an F-1 score of 0.97. Similarly, Bukhari et al. provide a comparative analysis of colonic adenocarcinomas using ResNet architectural alterations that reach a baseline accuracy of 93%. Wang et al. [41] suggested a cascaded classifier that detects mitotic cells by combining handcrafted features and those learned by CNN. Masud et al. [42] suggested an approach for detecting lung nodules utilizing CT scan images and a light CNN architecture. When evaluated on the LIDC dataset, their model achieved 97.9% classification accuracy when differentiating between normal, benign, and malignant cases. Shakeel et al. [43] proposed another process for lung cancer screening based on CT scan images.

## 3. Materials and Methods

**Figure 1:**
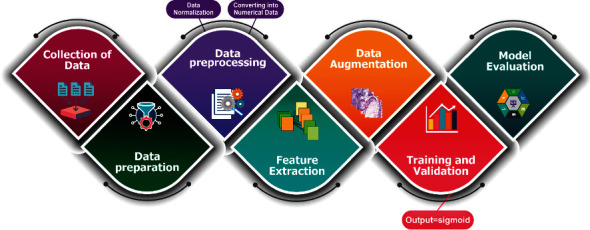
The proposed methodology's workflow.

We provide the following phases, and the flowchart of our process in this part shows in Figure 1. The classification task between benign and adenocarcinoma tissue is performed using DCNN and transfer learning models. Our suggested DCNN model includes some additional layers. Additionally, we compare the performance of many transfer learning models on this same dataset, including ResNet50, DenseNet121, ResNet101V2, EfficientNetB0, VGG-16, and MobileNetV2.

### 3.1. Details about the Dataset

From the LC25000 datasets, a total of 10 thousand digital photographs of histopathology slides were available. The collection contains histological images of osteosarcoma stained with hematoxylin and eosin (H&E). A team of clinical scientists at the University of Texas Southwestern Medical Center in Dallas gathered the data. This dataset was created from archival samples from 50 patients treated at Children's Medical Center, Dallas, from 1995 to 2015 [44]. It contains 500 images of colon tissue in total (250 images of benign colonic tissue and 250 images of colon adenocarcinomas), which have been augmented to 10,000 images using the Augmentor program [45]. Detailed contents of the dataset are shown in Table 1. Colon adenocarcinoma is the most frequent colon cancer, accounting for almost 95% of all cases. Adenocarcinoma develops when a specific type of polyp (tissue growth) called adenoma forms inside the large intestine and eventually transforms into cancer. All images in the dataset are HIPAA-compliant, verified, and royalty-free.

### 3.2. Preprocessing

Our goal in preprocessing is to create images that are suitable for the following step of the detection system. Preprocessing data are the first and most critical step in preparing data for use with a machine learning model. To get a high classification rate in our suggested study, we eliminated noise and artifacts from the images. Additionally, we did data reduction, data normalization, feature extraction, and ultimately, we turned the label string data to numerical data. Reduction is the mapping of a high-dimensional space to a lower-dimensional space that is more meaningful. In some instances, it is a crucial step prior to developing models.

### 3.3. Data Normalization

Data normalization is an approach to design a record that reduces data severance, decency, and undesirable features, for example, supplement, bring up to date, and removal variances. Several extant normalization approaches are present, including minmax normalization [46], z-score normalization [47], and decimal scale normalization [48]. We used z-score normalization to normalize our dataset according to the following formula:

(1) $$\begin{array}{c} V_{i}=\frac{Vi-Q\;}{\sigma Vi}, \end{array}$$

where *V*_i'_  is the Z-score normalized values and *v*_*i*_ is the value of row S of i-th column.

(2) $$\begin{array}{c} \text{Std}\left(V_{i}\right)=\sqrt{\frac{1}{\left(n-1\right)}}\sum_{i=1}^{n}{\left(V\text{i}-Q\right)}^{2}. \end{array}$$

It is both the concept and the action of putting disparate variables on an identical scale. This notion enables the comparison of scores for many sorts of factors. The basic idea behind this strategy is to change the data by converting it to a standard scale. The average number/mean equals zero, and the standard deviation equals one. It is a technical term that refers to the standard deviations below or above the mean.

### 3.4. Feature Extraction

The technique of feature extraction is critical in image processing because it divides the image into more manageable groupings for subsequent processing. We extract a significant number of characteristics in our research that aid in identifying and recognizing the pattern in a vast number of datasets [49]. Feature extraction is the process of converting given input data into a set of features [50]. In machine learning, feature extraction begins with a consistent collection of data and creates borrowed values, also known as features. These borrowed values, which are meant to be descriptive and nonredundant, simplify the subsequent learning and observation phases. In a few instances, it indicates improved human-kind analysis [51]. It is most closely related to dimensionality reduction. Principal component analysis (PCA) was employed to perform analysis on our image collection. PCA has a maximum number of relevant components that can be retrieved. Under particular signal and noise models, PCA-based dimensionality reduction tends.

To reduce such information loss,

(3) $$\begin{array}{c} x=\text{signal}+\text{noise,} \end{array}$$

where *x* defines the data vector of the desired information-bearing signal and noise.

Linsker [52] demonstrated, in particular, that if the signal is Gaussian and the noise is Gaussian noise with a covariance matrix corresponding to the identity matrix, the PCA algorithm maximizes the mutual reports between the required data and the output with decreased depth:

(4) $$\begin{array}{c} y=W_{L}^{T}x. \end{array}$$

Its works based on the following components:

1. Normalize the data: Unscaled data with different measurement units might affect the relative comparison of variance across features, so it is important to correct the data before running a PCA.
2. Creating covariance matrix for Eigen decomposition: Creating possible relationships between all of the different dimensions by understanding the total percentage of variance recorded by each primary component is crucial to decreasing the feature set.
3. Choosing the most appropriate amount of primary components: The cumulative explained variance ratio as a function of the number of principal components is used to estimate the appropriate number of it. The trade-off between dimensionality reduction and information loss determines which PCs are used.

## 4. Conversion into Numerical Data

In machine learning algorithms for accusation, numerical value is the most common sort of input to handle. We maintain a process to obtain numerical values with different scales for each attribute. Furthermore, these data must be simplified and regulated in order to improve training and model validation for a diverse set of DL control systems [53]. The LabelEncoder feature supplied by the python standard library is being utilized in our experiment to translate the two labels from benign to adenocarcinoma into 0 and 1.

### 4.1. Data Augmentation

Data augmentation is an approach for artificially increasing the amount of data by inserting slightly changed copies of current training data without actually gathering new data. You can manually increase the volume of the training dataset by either data distorting or oversampling, or you can help the model avoid overfitting from the start [54]. Data augmentation is a technique for reducing model overfitting in which we enhance the amount of training data by utilizing only the information contained in our training data [55]. We applied some augmentation parameters with suited values after splitting the dataset into training, testing, and validation sets by rotating, random cropping, mirroring, and color-shifting [56]. The factors we utilized to enrich our dataset are depicted in Table 2.

## 5. Our Proposed DCNN Model

**Figure 2:**
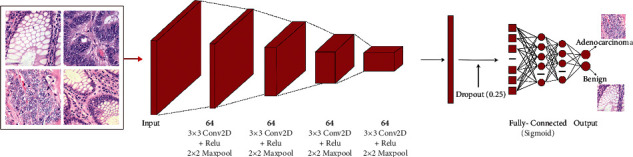
Our proposed DCNN model's architecture.

Transfer learning is the advance of learning in a new effort by transferring knowledge from an existing one [57]. Transfer learning is an ML technique that allows a model to be reused on related tasks after it has been trained [58]. Transfer learning (TL) is a method of conquer distant learning models and applying knowledge gained from one assignment to others. It is the task of using a pretrained system's information to learn new models from new data. Calibrating a pretrained system with TL is usually faster and easier. Pretrained DL systems help us quickly learn new occupations. Many scientists and data experts regard TL as a significant tool for accelerating AI development [59]. It is difficult to train a huge medical dataset like ImageNet utilizing all the neural network settings. However, we used a large medical dataset to train ResNet50, DenseNet121, EfficientNetB0, VGG-16, MobileNetV2, and our suggested DCNN model. Our suggested DCNN model used two classifications to distinguish cancer from benign colon tissue. The back-propagation function is used to fine-tune the updated weights. The Adam optimizer [60] is used in our suggested DCNN model. Finally, augmentation is used to overcome the dataset's labeled image constraints [61]. The structure of our suggested DCNN model is depicted in Figure 2. As illustrated in Algorithm 1, we have also suggested an algorithm for the classification of colon cancer.

### 5.1. Input layer

This layer loads needed data and feeds it to the first convolution layer. In our case, the input is a 128 × 128 pixel image with three color channels, which equals 3 for RGB.

### 5.2. Pooling layer

The pooling process is used to downsample the convolution layer's output images. It is used to compress the size of the input photos in order to facilitate training. All pooling layers employ the most widely used max pooling method.

### 5.3. Optimizer (Adam)

Adam is a stochastic gradient descent exchange optimization approach for minimizing the loss function associated with training DL models. We chose the standard gradient descent algorithm with a momentum of 0.999, and the learning rate was 0.001 for our investigation.

### 5.4. Flatten layer

This layer converts the output of the convolution layer to a 1D tensor, which is then used to connect a dense or fully connected layer.

### 5.5. Dropout layer

To avoid the model layers becoming overfit, we utilize a dropout layer between fully linked layers that randomly eliminates neurons from both visible and hidden levels.

### 5.6. Number of epochs (50)

DNN is passed over 50 times the dataset is used.

The parameters in our proposed DCNN model can be changed in a variety of ways. However, we've listed the most relevant ones we employed in our experiment (along with their values). In comparison with many other settings and deep neural network models that have been investigated earlier, this formation stands out [61].

**Figure 3:**
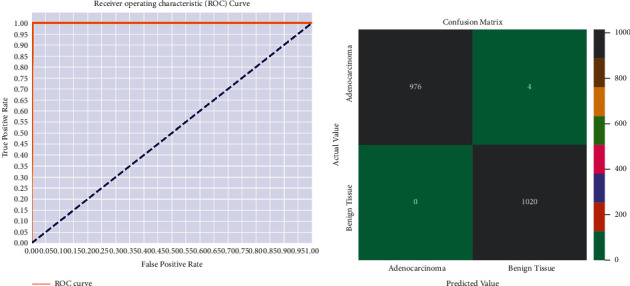
Our proposed DCNN model's ROC curve and confusion matrix. (a) Receiver operating characteristic (ROC) curve and (b) confusion matrix.

**Figure 4:**
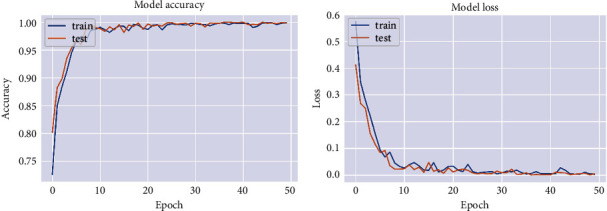
Our proposed DCNN model's performance curve in terms of accuracy and loss per epoch. (a) Accuracy curve and (b) loss curve.

The flat and progressive models were both recently developed, and the dataset was used to finely tune their insights into the categorization challenges of skin disorders. To finalize and assess the suggested DCNN model, we divide the dataset into three sets: training, validation, and testing. For a better comparison between models of transfer learning and our suggested model of DCNN, the dataset is divided into three sections: 80% training, 10% testing, and 10% validation set. Using train_test_split() from the scikit-learn data science toolkit, we split the dataset into subsets to reduce evaluation and validation bias. On the basis of accuracy, precision, recall, and Fmeasurement, each model is compared to the existing neural network models. The confusion matrix is also shown in Figure3(b). In addition, Figure 4(a) and Figure 4(b) depict the accuracy and loss between the training and testing phases of our proposed DCNN model, respectively.

## 6. Result and Discussion

The primary objective of our proposed model is to classify benign and adenocarcinoma colon cancer tissue retrieved using DCNN. When considering medical image processing, two metrics can be used to evaluate it. The first is at the patient level, that is, determining the number of correctly classified images for each patient. Second, it can be examined at the image level, where we calculate the percentage of correctly classified cancer images.

**Figure 5:**
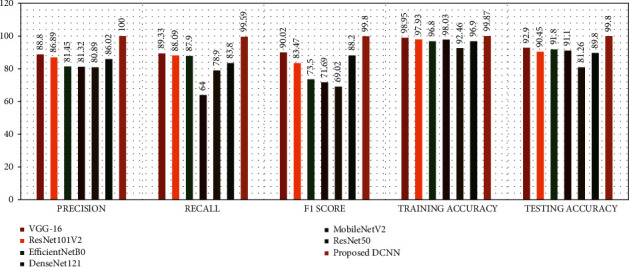
Performance comparison graph.

**Figure 6:**
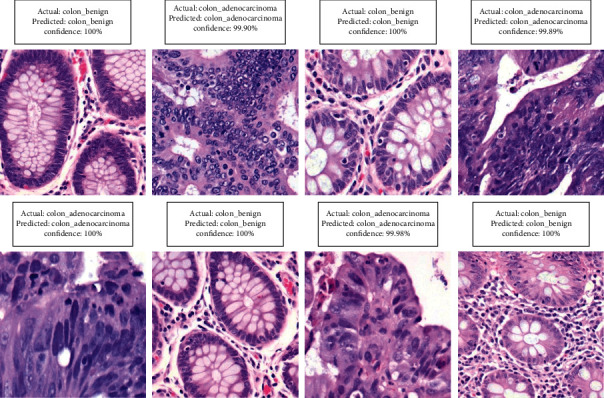
The prediction outcomes from our proposed DCNN model.

We divided the dataset into different portions when it could not achieve higher performances. To ensure that classifiers generalize effectively, we divided the data into three groups, with 80-10-10 of the data going into training, testing, and validation sets, respectively. Additionally, we ran several transfer learning models on the same dataset, including ResNet50, DenseNet121, ResNet101V2, EfficientNetB0, VGG-16, and MobileNetV2, yet our suggested DCNN model achieved the highest classification rate. By retraining these final few layers of transfer learning models on the premise that the extracted features are identical to those in the original application on which the network was trained, but the features are grouped differently in the new application [62].  Table 3 compares our proposed DCNN model to some transfer learning approaches. To facilitate comprehension, Figure 5 depicts the performance measurement. All CNN models were trained using the Tensor-Flow framework on Google's Colab [63]. The suggested DCNN model's results are compared to some existing works, which are shown in Table 4. There are a few limitations to achieving lesser performance, including a smaller number of input data, partitioning them into an appropriate portion, effective preprocessing methods, noise, and artifacts in images, and inappropriate hyper-parameter settings on their model. On the basis of numerical performance and visual findings, a full explanation of the suggested DCNN model is done. We also carried out our proposed model with 100 epochs; however, the model was overcompatible at the time. After multiple finetuning, we have achieved the best results at 50 epochs and achieved a curve area value of 0.998 (AUC), which is shown in Figure 3(a). DL approaches are advanced ML techniques that do not need to be designed by field specialists to extract features but learn by themselves. We can learn the functional detectors learned by models, considering the weights of feature maps. We iterated the process of fine-tuning our proposed model several times until arriving at the required values. The prediction outcomes from our proposed DCNN model are shown in Figure 6.  Table 5 compares the execution times of several transfer learning models and our proposed DCNN model. Compared to transfer learning models, the proposed model's architecture contains fewer layers, resulting in shorter execution times. We conducted this experiment by taking 10,000 images with two labels, demonstrating excellent performance. It may also perform well on a large dataset we intend to work on soon.

The suggested model uses parameter sharing and dimension reduction, significantly lowering calculations. The core premise is that learning from one area of an image can be applied to another, resulting in improved performance. Using the suggested DCNN model, a CAD system can efficiently classify colon cancer at an early stage. Furthermore, early detection of adenocarcinoma growth in the colon, particularly in persons who do not have access to a doctor, might greatly motivate them to seek treatment and improve their chances of survival.

### 6.1. Future Work

We plan to work on a larger dataset with more labeled colon tissue in the future to build up a successful DNN with preprocessing processes to provide the best accuracy in prediction and classification. Colon cancer can also be diagnosed with a convenient and hearty CAD for all acquired image circumstances. We will also try to create a DNN that can detect different sorts of skin lesions via CAD systems.

## 7. Clinical Relevance

Computer-aided detection and quantification, or CAD, is a well-established and constantly expanding field of study. The establishment of publicly accessible databases for training and validation is the most promising technique for improving CAD. It can help discover the most promising new research avenues and provide a platform for combining several approaches to produce superior algorithms for a single task [64]. The sensitivity of the CAD software was comparable to that of general radiologists, although there were more false positives. When utilized as a second reader, CAD detection of results incremental to radiologists shows benefit [65].

## 8. Conclusion

In our study, the suggested DCNN model outperforms previous transfer learning models capable of classifying benign and adenocarcinoma colon tissues by replacing the sigmoid function for binary classification in the output activation layer. We have also proposed a training and evaluation technique for the training of the CNN architecture so that these textured images are high resolution without transforming them into low-resolution images. In addition, the method proposed was evaluated on a dataset, in which we gained a superior level of training and testing accuracy to other models of transfer learning. To the best of our knowledge, we know of a previous work [30] carried out in categorizing the benign colon tissue with adenocarcinoma on the same dataset. We get 100% precision, 99.80% recall, 99.87% f1-score, and 99.80% accuracy, which is greater than that. Based on the findings of this investigation and previously described observations, we have a precision of greater than 6%. The development of computer-supported technology for diagnosing malignant tumors will give pathologists a substantial amount of support.

## Figures and Tables

**Algorithm 1:**
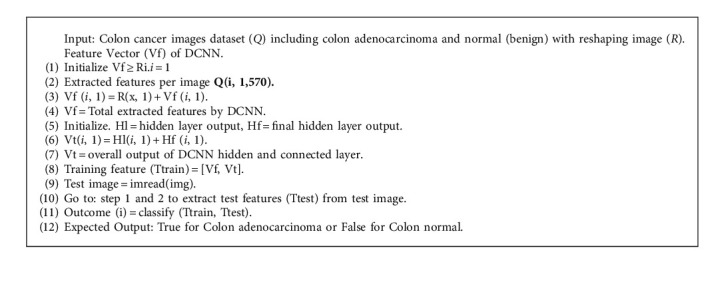
Proposed algorithm for the classification of colon cancer.

**Table 1:** The dataset's contents.

Cancer type	Class name	Number of samples
Colon adenocarcinoma	colon_aca	5000
Colon benign tissue	colon_n	5000

**Table 2:** Data augmentation parameters that we used in this study.

Parameter names for data augmentation	Parameter value	Action
Rotation range	360	With the rotation from—360 to 360, input data are generated.
Height shift range	0.2	Randomly moved image by 0.2.
Width shift range	0.2	At random, the image's horizontal position is shifted by 0.2.
Shear range	0.2	By a factor of 0.2, slant the image angle in degrees.
Zoom range	0.2	Zoom in or out by 0.2 distance from the middle
Horizontal flip	True	Randomly rotates the image horizontally
Fill mode	Nearest	The closest pixel value is used to fill the gaps

**Table 3:** Using our DCNN model to compare its performance with other transfer learning models.

Model	Precision	Recall	Fl score	Training accuracy	Testing accuracy
VGG-16	88.80	89.33	90.02	98.95	92.90
ResNet101V2	86.89	88.09	83.47	97.93	90.45
EfficientNetB0	81.45	87.90	73.50	96.80	91.80
DcnscNctl21	81.32	64	71.69	98.03	91.10
MobileNetV2	80.89	78.90	69.02	92.46	81.26
ResNet5O	86.02	83.80	88.20	96.90	89.80
Proposed DCNN	100	99.59	99.80	99.87	**99.80**

**Table 4:** Comparisons with previous work.

Reference	Cancer type	Image type	Classifier	Accuracy (%)	Precision (%)	Recall (%)	F-measure (%)
[41]	Colon	Histopathological	SVMs	—	73.7	68.2	70.8
[43]	Colon	Histopathological	SC-CNN	—	78.3	82.7	80.2
[32]	Colon	Histopathological	RI′	99		94	—
[3]	Colon	Colonoscopy	AlexNet	91.47	—	91.76	—
[47]	Colon	Histopathological	RI′	85.3	—	—	85.2
[21]	Colon	Colonoscopy	Faster R-CNN	98.5	100	98.5	99.24
[22]	Colon	Colonoscopy	CNN	96.4	—	93	—
[1]	Colon	Colonoscopy	CNN	90.28	74.34	68.32	71.2
[30]	Colon	Histopathological	RESNET-50	93.91	95.74	96.77	96.26
[29]	Colon	Histopathological	CNN	96.61	—	—	—
Proposed	Colon	Histopathological	DCNN	99.80	100	99.59	99.80

**Table 5:** Time comparison between our proposed DCNN and transfer learning models.

Model	Time per epochs (seconds)	Total time (minutes)
VGG-16	24 ± 2	22 ± 1
EfficientNetB0	19 ± 2	19 ± 1
ResNet101V2	20 ± 2	19 ± 1
ResNet5O	21 ± 2	18 ± 1
DenseNet121	18 ± 2	16 ± 1
MobileNetV2	17 ± 2	16 ± 1
Proposed DCNN	9 ± 2	9 ± 1

## Data Availability

The data used to support the findings of this study are available from the corresponding author upon request.
